# Erratum

**DOI:** 10.1093/jisesa/iex012

**Published:** 2017-03-24

In “Impact of pesticide resistance on toxicity and tolerance of hostplant phytochemicals in *Amyelois transitella* (Lepidoptera: Pyralidae)”, the title included an error: the second word in the species name was capitalized. This has now been fixed in the original article.

## References

[iex012-B1] Bagchi V. A. Siegel J. P. Demkovich M. R. Zehr L. N. Berenbaum M. R. 2016 Impact of pesticide resistance on toxicity and tolerance of hostplant phytochemicals in *Amyelois transitella* (Lepidoptera: Pyralidae). J. Insect Sci. 16: 62. doi: 10.1093/jisesa/iew063. 10.1093/jisesa/iew063 PMC5019020 27620560

